# Anal Melanoma in an Elderly Woman Masquerading as Hemorrhoid

**DOI:** 10.7759/cureus.1880

**Published:** 2017-11-26

**Authors:** Gabriel O Ologun, Yuan Stevenson, Alice Shen, Navpreet K Rana, Amber Hussain, David Bertsch, Burt Cagir

**Affiliations:** 1 General Surgery, Robert Packer Hospital/Guthrie Clinic; 2 Surgical Oncology, Robert Packer Hospital/Guthrie Clinic; 3 Colorectal Surgery, Robert Packer Hospital/Guthrie Clinic

**Keywords:** anus, melanoma, surgery, wide local excision, abdominoperineal resection, transanal excision

## Abstract

Anal melanoma is a rare and aggressive neoplasm of the anal canal seen in the elderly population in the six or seventh decade of their lives. Presentation is usually nonspecific and diagnosis is often delayed or missed initially. The management is surgical and prognosis is poor. Here we present a case of anal melanoma in an elderly patient masquerading as hemorrhoid.

## Introduction

Anal melanoma is a rare and aggressive neoplasm of the anal canal, comprised about 2% of anal neoplasm cases identified using the Surveillance Epidemiology and End Results (SEER) registry [1]. Females are more likely to be affected than males and most patients present in the sixth or seventh decade of their lives [2]. Patients typically present with nonspecific symptoms of rectal bleeding, pruritus, pain, and anorectal mass that mimics other benign conditions such as hemorrhoid, rectal polyp, rectal prolapse, resulting in misdiagnosis or delay in diagnosis with an average duration of symptoms before diagnosis between four and six months [3,4]. Here we present a case of anal melanoma in an elderly patient masquerading as hemorrhoid. Informed consent was obtained for the case report, images, and for publication.

## Case presentation

An 88-year-old woman with history of hypertension, hyperlipidemia, coronary artery disease, constipation, sedentary was referred by her primary care provider to an outside general surgery clinic with diagnosis of bleeding hemorrhoid. The patient had four weeks history of anal discomfort with defecation, sensation of protruding mass in the anal region and intermittent bleeding from her anus. No prior colonoscopy. Evaluation of the anus at that time revealed a prolapsing purple-black mass below the dentate line thought to be a thrombosed hemorrhoid. Local excision was recommended at that time however patient and daughter declined surgery because her pain had been improving and her bowel habits had been regular. She would consider surgical intervention if her symptoms got worse.

**Figure 1:**
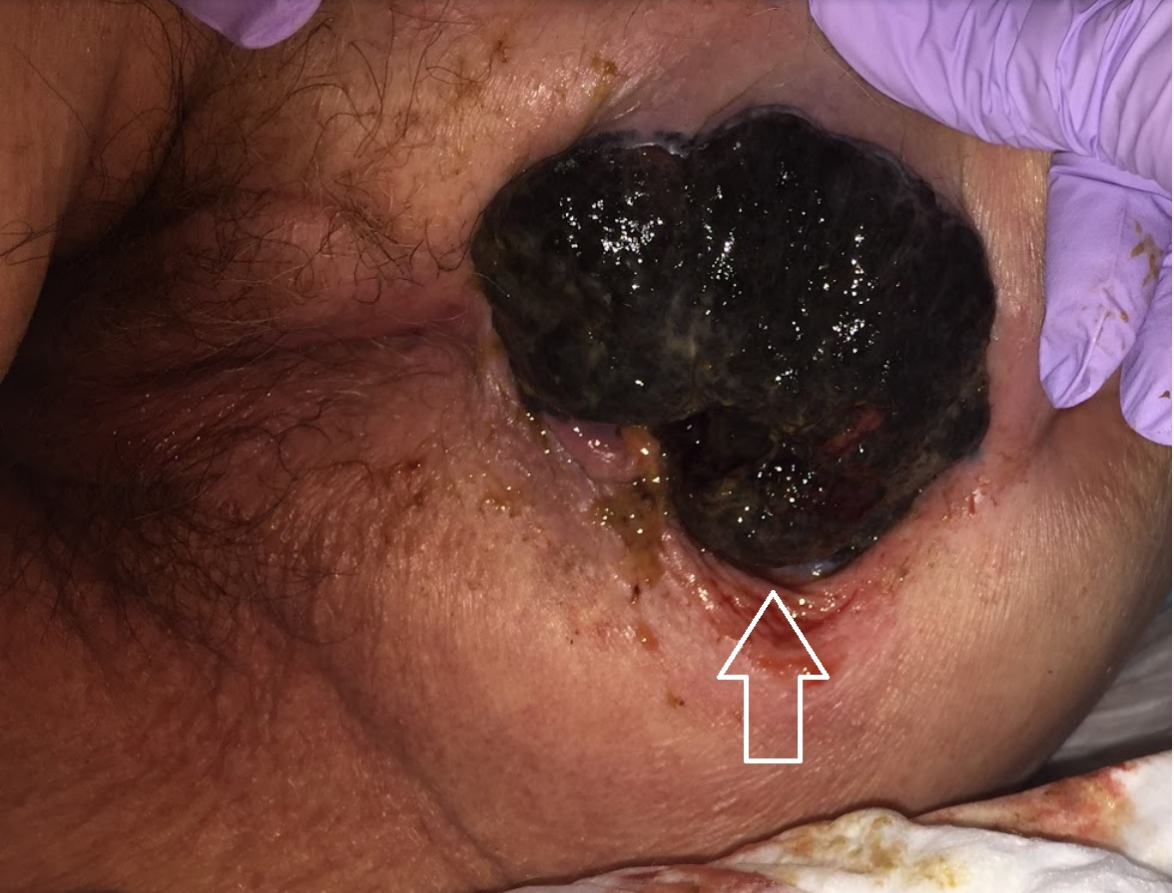
Anal melanoma (arrow).

The patient presented, 10 weeks later, to an outside emergency department with a complaint of enlarging perianal mass and pain, worse with seating. Surgical service was consulted. On exam she was noted to have a necrotic appearing pigmented lobulated anal mass measuring 7 cm x 5 cm. Biopsy was obtained and the patient was admitted to the surgical service. The result of the biopsy revealed malignant melanoma, ulcerated, invasive to a depth of 4.1 mm. She was then transferred to our hospital for management. Computed tomography (CT) scan of the chest, abdomen and pelvis with contrast showed perianal mass with left inguinal lymphadenopathy. The patient and daughter opt for palliative management. She was taken to the operating room and underwent exam under anesthesia, and a palliative wide local excision (WLE) was performed. The total excised tissue was 7.3 cm x 6.2 cm x 2.7 cm irregular dark brown mass (Figure 1).

Pathologic examination revealed malignant melanoma, invasive to a depth of at least 19 mm, with extensive lymphovascular invasion and tumor involving the deep and radial margins of the specimen. She recovered well from the surgery, and was discharged to her personal home care facility on postoperative day three with home hospice care.

## Discussion

The prognosis for anal melanoma is poor, with overall five-year survival rate of 10-20% [5,6], with a median survival of less than 20 months [3]. Hence any atypical anorectal lesion should be biopsied in order to prevent delay in diagnosis [6].

About 20% of patients with newly diagnosed anal melanoma have lymph node-positive disease in the inguinal region [3]. The proportion of patients with systemic metastasis at presentation ranges from 7% to 25% [3]. Immunohistochemical staining can be useful in workup of a suspicious anorectal lesion. Melanoma is positive for S-100 protein, HMB-45, and vimentin. It is negative for carcinoembryonic antigen (CEA), cytokeratin, and epithelial membrane antigen. In patients diagnosed with anal melanoma, it is reasonable to rule systemic disease by evaluating with CT scan of head, chest, abdomen and pelvis [3].

The management of anal melanoma traditionally has included abdominoperineal resection (APR). However, there has been a trend in favor of less aggressive WLE due to the high morbidity of APR and perceived lack of survival benefit [3,6].

Survival after WLE is similar to abdominoperineal resection, which has prompted tertiary oncology centers to recommend transanal excision over radical surgery in most cases. The result of systemic therapy for anal melanoma is disappointing. Radiation alone provides the option of sphincter preservation but does not increase survival [3,6,7].

## Conclusions

Anal melanomas continue to be diagnostic challenge due to their nonspecific symptoms making early diagnosis extremely difficult. Early diagnosis and early surgical intervention are imperative. It is associated with poor prognosis, regardless of the type of intervention used. The most important role of the surgeon may be palliation of symptoms.
